# Plasma miR-323 as a Biomarker for Screening Papillary Thyroid Cancer From Healthy Controls

**DOI:** 10.3389/fmed.2020.00122

**Published:** 2020-05-15

**Authors:** Yang Liu, Lin Li, Zheng Liu, Qingling Yuan, Xiubo Lu

**Affiliations:** ^1^Department of Thyroid Surgery, The First Affiliated Hospital of Zhengzhou University, Zhengzhou, China; ^2^Department of Dermatology, Henan Children's Hospital, Zhengzhou, China

**Keywords:** plasma miR-323, papillary thyroid cancer, metastasis, biomarker, fine-needle aspiration cytology

## Abstract

The present study aims to evaluate whether plasma miR-323 serves as a potential biomarker to screen patients with papillary thyroid cancer (PTC) from healthy controls. Real-time PCR was performed to evaluate miR-323 expression in healthy controls and benign thyroid nodule (BTN) and PTC patients. Receiver operating characteristic (ROC) curve analysis was used to evaluate whether plasma miR-323 could be used to screen PTC patients from BTN patients and healthy controls. Plasma miR-323 was significantly increased in PTC patients compared with that in BNT patients and healthy controls. Moreover, miR-323 in the thyroid tissue was significantly increased in PTC patients when compared to BNT patients. We further showed that plasma and tissue miR-323 levels were significantly increased in PTC patients with metastasis compared to those without metastasis. Plasma miR-323 was significantly increased in PTC patients with BRAF V600E mutation when compared to those with wild-type BRAF. Furthermore, plasma miR-323 was significantly increased in PTC patients with higher Tg-FNAB. ROC analysis showed that plasma miR-323 could distinguish PTC patients from BNT patients and healthy controls. The present study demonstrated that plasma miR-323 might be an effective noninvasive indicator for PTC progression and serve as a biomarker for the diagnosis of PTC.

## Introduction

Thyroid cancer is the most common malignant endocrine tumor, and its prevalence is rapidly increasing worldwide (1). Approximately 300,000 new patients are diagnosed each year, with around 40,000 deaths per year (1, 2). Papillary thyroid cancer (PTC), a differentiated neoplasia, is the most prevalent type among all thyroid cancers (3). It is of clinical importance to distinguish PTC from benign thyroid nodules (BTNs), including thyroid adenoma and classical nodular goiter. Currently, ultrasound and computed tomography (CT) are routinely used to examine patients with suspicious thyroid nodules (4), after which preoperative ultrasound-guided fine-needle aspiration cytology (FNAC) and intraoperative pathological examination are subsequently employed to examine the patients with suspicious PTC (4). FNAC serves as an efficient, cost-effective, and reliable diagnostic tool with remarkable sensitivity and relative low specificity for the initial screening of patients with thyroid nodules (5). As an invasive approach with potential false negative and false positive, the diagnostic efficacy and accuracy of FNAC in PTC patients were compromised with various influencing factors such as the thyroid nodule size (6) and the misinterpretation of cytopathological results due to the morphological overlap between PTC in chronic lymphocytic thyroiditis and pure thyroiditis (7, 8). Nonetheless, these observations necessitate the development and validation of novel non-invasive approaches to improve the disease detection in early stage and prediction of disease prognosis in PTC patients (9). Recently preoperative molecular analysis using a panel of genetic alterations emerges as a new approach to make up the limitation of FNA diagnosis (10). Therefore, it is of clinical importance to explore noninvasive biomarkers to screen patients from individuals with benign thyroid lesions.

MicroRNAs are small noncoding RNAs with approximately 22 nucleotides (11). Through base pairing mechanism, miRNAs are extensively involved in the regulation of various biological and pathological processes, including cell proliferation, differentiation, and death (12–14). Currently, accumulating studies have identified various miRNAs as ideal biomarkers since they stably present in the serum and plasma and can be detected with high sensitivity and specificity (15, 16). For instance, miR-126-3p serves as a tumor suppressor to inhibit thyroid cancer cell growth and metastasis (17). miR-375 suppresses thyroid carcinoma cell proliferation and induce cell apoptosis *via* binding the 3′ untranslated region of ERBB2 (14). Meanwhile, miR-222 and miR-146b are positively correlated with the progression of PTC in the tissue and serum of patients with recurrent PTC (18). It was recently reported that miR-323 was dysregulated in prostate cancer and pancreatic ductal adenocarcinoma (19, 20), while little is known about its role in the progression of PTC. The current study aimed to evaluate the correlation of miR-323 with PTC progression and its potential role as a biomarker to screen patients with PTC from healthy controls.

## Materials and Methods

### Patients

This study protocol was approved by the Medical Institutional Ethics Committee of the First Affiliated Hospital of Zhengzhou University. A total of 100 patients with primary PTC, 50 patients with BTNs, and 20 age- and gender-matched healthy controls from the First Affiliated Hospital of Zhengzhou University were enrolled in this study from March 2015 to December 2016. Written informed consent was obtained from all participants. The formalin-fixed and paraffin-embedded (FFPE) PTC tissues or BTN tissues were used for the postoperative histopathologic diagnosis and miRNA examination. The freshly isolated samples were immediately frozen for preparing total RNA. In addition, blood samples were isolated from all subjects before surgery and were also collected from six patients after tumor resection and radiometabolic therapy for 2 weeks after surgery. The details of the clinical features are shown in Table 1.

**Table 1:** Clinical features of PTC patients and healthy controls.

**Variable**	**PTC patients**	**BTN patients**	**Healthy controls**
Male/female	53/47	22/28	9/11
Age (year)	54.3 ± 10.3	48.9 ± 15.1	55.7 ± 8.7
Tumor size (cm)			
≤ 1	56	–	–
>1	44	–	–
Capsular invasion			
Yes	32	–	–
No	68	–	–
Lymph node metastasis			
Yes	35	–	–
No	65	–	–
No. of cancer foci			
Single	56	–	–
Multiple	44	–	–
BRAF^V600E^ gene			
Mutant	72	–	–
Wide type	28	–	–

### US-Guided Fine Needle Aspiration Biopsy (FNAB) Cytology of Thyroid Nodules

FNAB was carried out by endocrinologists using a 25-gauge needle. After each pass, the cytological material was immediately smeared onto slides. The slides were prepared by both air-dried and alcohol-fixed methods. The air-dried smears were stained with the Diff-Quik method for an immediate on-site evaluation, whereas the alcohol-fixed smears were stained with the Papanicolaou method in the cytological laboratory.

A cytopathologist performed an on-site evaluation after each FNA pass. Thereafter, the material from the Diff-Quik smears was deemed arbitrarily as “adequate” (sufficient lymphocytes), “less than optimal” (some lymphocytes), or “inadequate” (a very few or no lymphocytes); a dedicated FNA pass was performed without smears, and the FNA needle was rinsed in a tube containing 1 ml of Hank's balanced salt solution without heparin. Specimens were immediately transferred to the clinical laboratory and stored at −20°C for 0 to 4 days before thyroglobulin (Tg) analysis. For each case, all passes were performed by the same endocrinologist.

### Tg-FNAB Antibody Assays

The measurement of Tg levels in needle washouts (Tg-FNAB) was carried out with a commercial immunofluorometric assay using monoclonal antibodies (DELFIA^®^, PerkinElmer, Turku, Finland), with a functional sensitivity of 1.0 ng/ml.

### Sample Acquisition and RNA Extraction

Plasma miR-323 was evaluated at first diagnosis, prior to any treatment, in all involved participants, unless specified (e.g., follow-up study). A 5-ml aliquot of blood was collected from each participant directly into sodium citrate tubes. Ten micrograms of freshly isolated samples with remarkable lesions from PTC or BTN patients were processed in lysis buffer at 4°C and proceeded to further miRNA analysis. Total RNA from plasma samples and freshly isolated tissue samples was extracted with RNAVzol LS (Vigorous, Beijing, China) according to the specific instructions for small RNA isolation. The quality, quantity, and integrity of RNA were determined using a NanoDrop spectrophotometer (ND-1000, Nanodrop Technologies).

### qPCR Validation

RNA was reverse transcribed into cDNA with the Prime-Script one-step qRT-PCR kit (C28025-032, Invitrogen). Detailed qRT-PCR procedure was performed as follows: 95°C for 10 min followed by 50 cycles at 95°C for 10 s, 55°C for 10 s, and 72°C for 5 s; 99°C for 1 s; 59°C for 15 s; 95°C for 1 s; and then cooling to 40°C. The relative expression levels were calculated with the 2^−ΔΔCt^ method and experiments were repeated in triplicate. The specific primers used in the current study were listed as follows:

miR-323-RT: GTCGTATCCAGTGCAGGGTCCGAGGTATTCGCACTGGATACGAGCGAA;
U6-RT: GTCGTATCCAGTGCAGGGTCCGAGGTATTCGCACTGGATACGACAAATATG;
miR-323-F: GCGCAGGTGGTCCGTGGCGCG;
U6-F: GCGCGTCGTGAAGCGTTC;
Universe reverse primer: GTGCAGGGTCCGAGGT.

### BRAFV600E Gene Testing

The determination of BRAFV600E gene mutation was carried out for all PTC patients by PCR techniques. DNA was isolated using a DNA extraction kit (Promega Corporation, CA, USA), and the BRAF gene exon 15 was detected using a BRAF mutant gene detection kit (Amoy Diagnostics Co., LTD, China) on an ABI7500 real-time PCR amplifier (Promega Corporation, CA, USA). The primers used for the amplification of BRAF exon 15 were presented as follows: forward (5′-TCATAATGCTTGCTCTGATAGGA-3′) and reverse (5′-GGCCAAAAATTTAATCAGTGGA-3′). All procedures and analyses were performed in the institutional biomolecular laboratory.

### Statistics

The data are represented as mean ± standard error (SD). Two-tailed unpaired Student's *t*-tests were used for comparisons of two groups. ANOVA multiple comparison test (SPSS 13.0) followed by Tukey's *post hoc* test were used for comparisons of two and more groups. Receiver operating characteristic (ROC) curves were used to assess miR-323 as a biomarker, and the area under the curve (AUC) was reported (version 20.0, SPSS, Inc., Chicago, Illinois). *p* < 0.05 was considered significant.

## Results

### Increased Plasma miR-323 Level in PTC Patients

**Figure 1:**
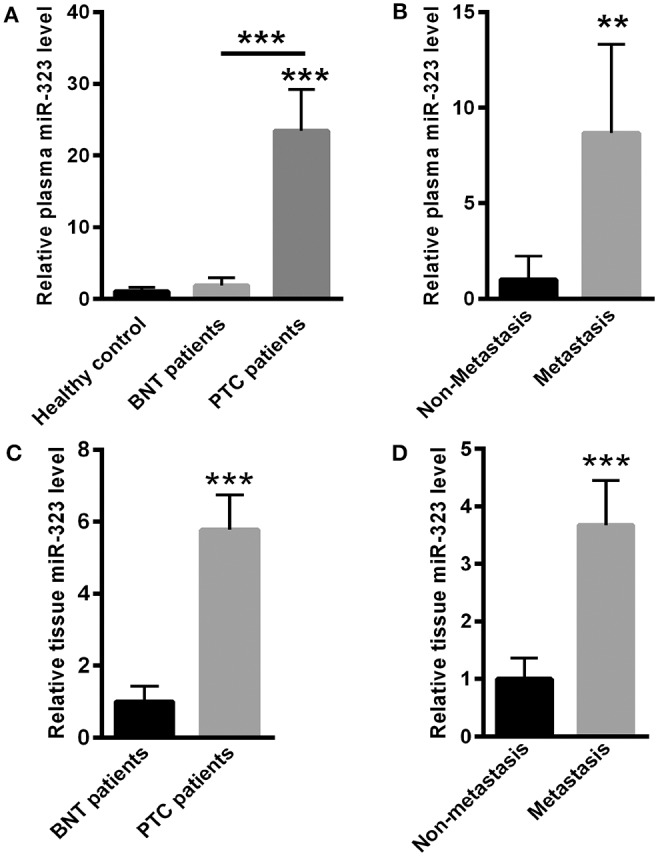
miR-323 levels were increased in the plasma and thyroid tissues of PTC patients. **(A)** Compared with healthy controls, plasma miR-323 levels were significantly increased in PTC patients, but not in BNT patients. **(B)** Plasma miR-323 levels were significantly increased in metastatic PTC patients compared to nonmetastatic PTC patients. **(C)** Real-time PCR analysis indicated that miR-323 was increased in the thyroid tissues of PTC patients compared to those in BNT patients. **(D)** miR-323 was increased in the tissues of metastatic PTC patients compared to those of nonmetastatic PTC patients. ^**^p < 0.01, ^***^p < 0.001 vs. as indicated.

First, we evaluated the level of miR-323 in PTC patients, BTN patients and healthy controls. Compared with the results for healthy controls, the plasma level of miR-323 was significantly increased in PTC patients, but not in BNT patients (Figure 1A). Furthermore, we also compared the plasma level of miR-323 in nonmetastatic and metastatic patients with PTC. Our data showed that miR-323 was significantly increased in metastatic PTC patients when compared to nonmetastatic PTC patients (Figure 1B).

### miR-323 Was Increased in the Tissues of PTC Patients

Next, we isolated RNA from tissues of PTC patients and BNT patients. Real-time PCR analysis indicated that miR-323 was significantly increased in the tissues of PTC patients when compared with those in BNT patients (Figure 1C). Meanwhile, our data also showed that miR-323 was increased in the tissues of metastatic PTC patients when compared to those of nonmetastatic PTC patients (Figure 1D).

### miR-323 Was Increased in the Plasma and Tissues of BRAF^V600E^ Mutant PTC Patients

**Figure 2:**
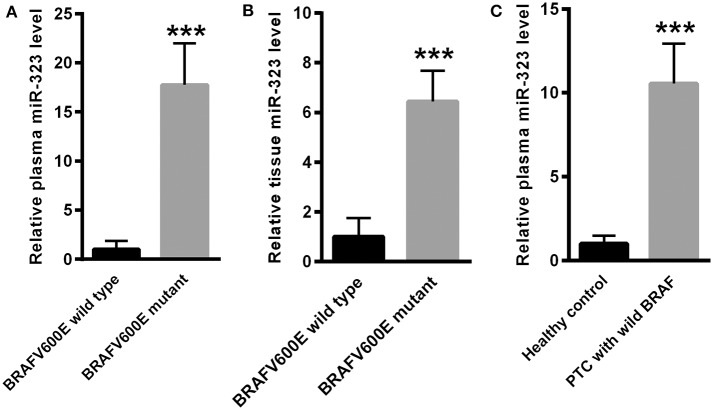
miR-323 was increased in the plasma and tissues of BRAF^V600E^ mutant PThC patients. **(A)** Compared with BRAF^V600E^ wild-type PTC patients, miR-323 was significantly increased in the plasma of BRAF^V600E^ mutant PTC patients. **(B)** miR-323 was significantly increased in thyroid tissues of BRAF^V600E^ mutant PTC patients compared with those of BRAF^V600E^ wild-type PTC patients. **(C)** Plasma miR-323 level was significantly increased in PTC patients with wild-type BRAF^V600E^ when compared to healthy controls **(C)**. ^***^p < 0.001 vs. as indicated.

Compared with wild-type BRAF^V600E^ PTC patients, plasma miR-323 was significantly increased in the plasma of BRAF^V600E^ mutant PTC patients (Figure 2A). We further compared the levels of miR-323 in the tissues of BRAF^V600E^ wild-type PTC patients and BRAF^V600E^ mutant PTC patients. Our data showed that miR-323 was significantly increased in the thyroid tissues of BRAF^V600E^ mutant PTC patients compared to those of wild-type PTC patients (Figure 2B). We also found the increased plasma miR-323 level in PTC patients with wild-type BRAF^V600E^ when compared to healthy controls (Figure 2C).

### miR-323 Levels Positively Correlated With Tg-FNAB Levels

**Figure 3:**
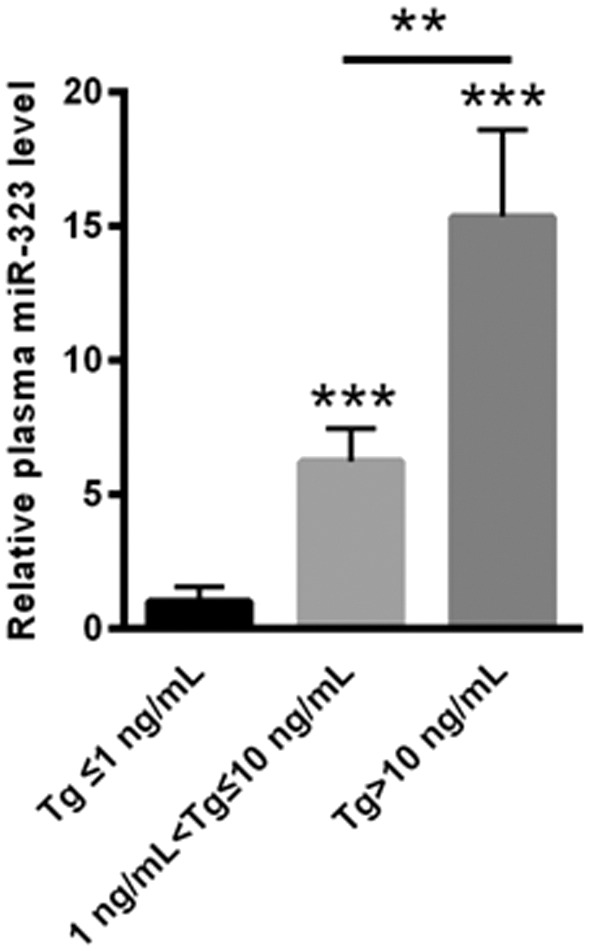
The level of plasma miR-323 was lower in PTC patients with Tg-FNAB ≤ 1 ng/ml, while it was much higher in thyroid lymph nodes of PTC patients with 1 ng/ml < Tg-FNAB ≤ 10 ng and Tg-FNAB >10 ng/ml. ^**^p < 0.01, ^***^p < 0.001 vs. as indicated.

**Figure 4:**
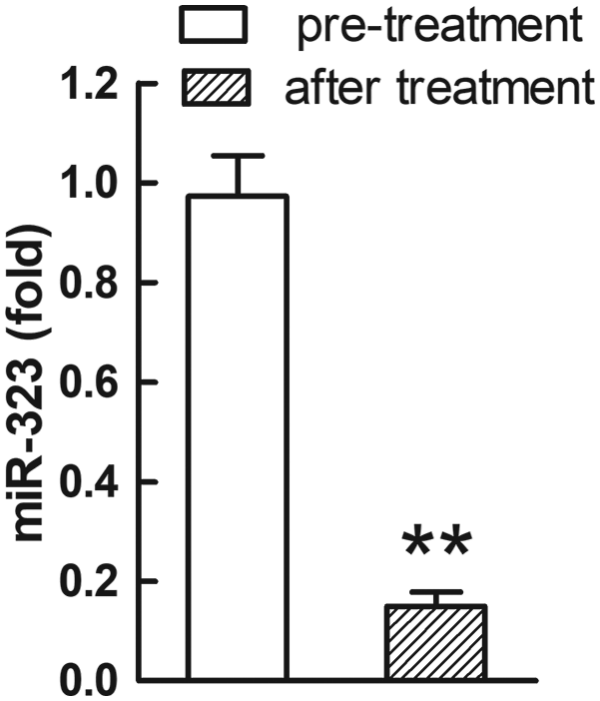
Plasma miR-323 levels were decreased in PTC patients after surgery and proper therapy. ^**^p < 0.01, *N* = 6.

The measurement of Tg in needle washouts (Tg-FNAB) is an important confirmation of malignancy in lymph nodes (LNs). Hence, we compared the level of plasma miR-323 among the groups of differential concentration of Tg-FNAB. As shown in Figure 4, the level of plasma miR-323 was lower in PTC patients with Tg-FNAB ≤ 1 ng/ml, while it was significantly increased in PTC patients with Tg-FNAB >1 ng/ml. We further found that plasma miR-323 in PTC patients with Tg-FNAB >10 ng/ml was significantly higher in those patients with 1 ng/ml < Tg-FNAB ≤ 10 ng/ml (Figure 3).

### Plasma miR-323 Was Decreased in PTC Patients After Surgery and Appropriate Therapy

We also carried out a follow-up assay for six PTC patients after they received appropriate treatment for 2 weeks. After surgery and two-week therapy, the relative plasma level of miR-323 was significantly decreased in the six PTC patients (*p* < 0.01, Figure 4).

### miR-323 Could Be Used to Screen PTC Patients From Healthy Controls

**Figure 5:**
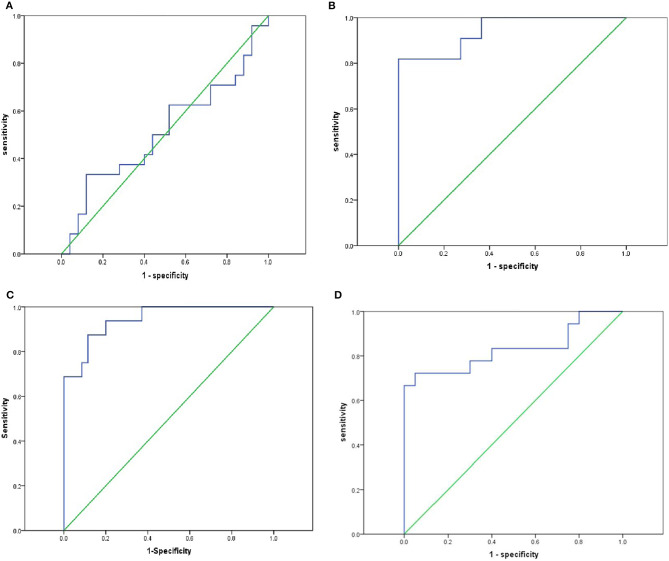
Plasma miR-323 could be a potential biomarker to screen PTC patients from healthy controls and BNT patients. **(A)** ROC analysis showed that plasma miR-323 levels failed to screen BNT patients from healthy controls. **(B)** ROC analysis showed that plasma miR-323 levels could distinguish PTC patients from healthy controls. **(C)** ROC analysis showed that plasma miR-323 levels could distinguish BNT patients from PTC patients. **(D)** ROC analysis showed that plasma miR-323 levels could distinguish the nonmetastatic PTC patients from metastatic PTC patients.

We evaluated whether miR-323 could be used as a potential biomarker to screen PTC patients from healthy controls. As shown in Figure 5A, while plasma miR-323 failed to distinguish BNT patients from healthy controls, as shown by the ROC curve area of 0.515 (95% confidence interval: 0.349–0.681; *p* = 0.857, Figure 5A), it could distinguish PTC patients from healthy controls, as shown by the ROC curve area of 0.942 (95% confidence interval: 0.858–1.000; *p* < 0.001, Figure 5B). Meanwhile, plasma miR-323 could also distinguish BNT patients from PTC patients, as shown by the ROC curve area of 0.912 (95% confidence interval: 0.819–1.000; *p* < 0.001, Figure 5C). In addition, plasma miR-323 was shown to distinguish nonmetastatic PTC patients from metastatic PTC patients, as shown by the ROC curve area of 0.831 (95% confidence interval: 0.688–0.973; *p* < 0.001, Figure 5D). These data suggested that plasma miR-323 levels could serve as a potential biomarker to screen PTC patients from healthy controls and BNT patients.

## Discussion

Currently, it is important to identify potential molecular predictors of PTC patients (21, 22). BRAF mutation is indicated as a potential marker of aggressive PTC, but it mainly depends on invasive fine-needle aspiration diagnosis (23). In addition, p27, p21, cyclin D1, CEACAM-1 (carcinoembryonic antigen-related cell adhesion molecule 1), osteopontin, and E-cadherin are also reported as potential biomarkers (24, 25), which are still far from clinical applications. The present study demonstrated that miR-323 was significantly increased in the plasma and thyroid tissue in PTC patients, and its expression was correlated with metastasis, BRAFV600E mutant, Tg-FNAB, and appropriate therapy. It can serve as a potential biomarker to distinguish PTC patients from BNT patients and healthy controls.

Abnormal expression of miRNAs has been widely identified in both papillary and follicular thyroid cancer (FTC) tissues (26–28). For instance, miR-144-3p is reported to increase the growth of tumor and the metastasis of PTC *via* suppressing the expression of PAX 8, which may be a promising prognosis marker and valuable treatment strategy for PTC (26). In addition, reduced miR-199a-5p expression is found in PTC tissues, and further study demonstrated that SNAI1 was a target gene of miR-199a-5p (27). The rs2910164 genetic variant of miR-146a-3p is also reported to be correlated with enhanced overall mortality in FTC patients (28). Additionally, miR-323 was significantly increased in patients with hereditary MTC (hMTC) and sporadic MTC (sMTC) (29). A previous *in vitro* study reported a decreased miR-323 in PTC cell line with BRAF mutations when compared to normal thyroid cell line (30). In the current study, we evaluated the level of plasma miR-323 in healthy controls, BNT patients, and PTC patients. We confirmed that plasma miR-323 of PTC patients was significantly increased compared with that of BNT patients and healthy controls. Meanwhile, thyroid tissue levels of miR-323 were strongly increased in PTC patients compared to BNT patients. The above observations suggested an oncogenic role of miR-323 in the progression of PTC.

A high survival rate has been found in patients with well-differentiated thyroid cancer (31). It is well recognized that the progression of metastasis is still the major cause of thyroid cancer-related mortality (32). Hence, it is important to identify genes related to this process, thereby identifying novel candidates for diagnosis or therapeutic intervention (33). Here, our data showed that plasma and tissue miR-323 levels were significantly increased in PTC patients with metastasis when compared to nonmetastatic patients.

The T1799A nucleotide transversion in the *BRAF* gene is an important oncogenic mutation linked to PTC (34, 35). It has been reported that 45% of PTC patients are carrying another mutation characterized by a valine-to-glutamic acid change in codon 600 of the BRAF protein (BRAF V600E), which increases the serine/threonine protein kinase activity of BRAF and thereby constitutively activates the mitogen-activated protein kinase signaling pathway (36, 37). Hence, the BRAF V600E is a potential key prognostic marker for PTC (36, 37). Our current results showed that plasma miR-323 level was significantly increased in PTC patients with BRAF V600E mutation compared to those with wild-type BRAF, while plasma miR-323 level in PTC patients with wild-type BRAF^V600E^ was also significantly increased when compared to healthy controls. This likely reflects that elevated plasma miR-323 is potentially associated with the more aggressive clinicopathological behavior of BRAF V600E mutation PTC.

In the clinic, the application of Tg-FNAB is well acknowledged as an important method to examine suspicious LN metastases (38, 39). However, 5–10% of FNABs are nondiagnostic, and 6–8% are false negatives (40). Hence, we explored the differential plasma miR-323 levels in PTC patients with different concentrations of Tg-FNAB. Compared with PTC patients with Tg-FNAB ≤ 1 ng/ml, plasma miR-323 levels were significantly increased in PTC patients with Tg-FNAB >1 ng/ml. This finding suggests that the analysis of plasma miR-323 levels could serve as an implemental invasive diagnostic method in line with Tg-FNAB levels, in which the accuracy of the Tg-FNAB method could increase when combined with analysis of plasma miR-323 levels.

ROC analysis was also employed to further evaluate whether miR-323 could be used as a potential biomarker to screen PTC patients from BNT patients and healthy controls. Our data showed that analysis of plasma miR-323 levels could effectively differentiate PTC patients from BNT patients and healthy controls.

*In silico* analysis with Targetscan software was applied to predict the potential targets of human miR-323, which revealed 488 transcripts with conserved sites as shown in Supplementary Table 1. Actually, multiple proteins and signaling pathways [e.g., p73 (19), insulin-like growth factor 1 receptor (41), and BRI3 (42)] were previously proposed to mediate the potential role of miR-323 in the pathogenesis and progress of various types of neoplasia. For instance, miR-323-3p suppressed the expression of transmembrane protein with EGF-like and 2 follistatin domain (TMEFF2) and the activation of AKT and ERK pathways, by which it inhibited the apoptosis in non-small-cell lung cancer (NSCLC) cell lines (43).

While the current study demonstrated for the first time that plasma miR-323 may be a validated noninvasive indicator for PTC progression, some limitations exist. For example, the molecular mechanism underlying miR-323 involvement in PTC progression need to be elucidated. Meanwhile, further study with a larger case population is also in need to validate the efficacy of plasma miR-323 and its correlation with serum/tissue Tg as a biomarker to evaluate the tumor progression, prognosis, and recurrence in clinical patients with PTC.

## Data Availability Statement

The datasets generated for this study are available on request to the corresponding author.

## Ethics Statement

The studies involving human participants were reviewed and approved by Medical Institutional Ethics Committee of the First Affiliated Hospital of Zhengzhou University. The patients/participants provided their written informed consent to participate in this study.

## Author Contributions

YL and XL designed the study. YL, LL, ZL, and QL collected the data. YL, LL, and XL analyzed the data. YL and XL wrote the manuscript.

## Conflict of Interest

The authors declare that the research was conducted in the absence of any commercial or financial relationships that could be construed as a potential conflict of interest.

## Supplementary Material

Supplementary Table 1 Clinical features, including gender, tumor size, capsular invasion, lymph node metastasis, number of cancer foci, and BRAF nutation, were presented in healthy controls and PTC and BTN patients.
